# The double-pedicled dorsal-metacarpal-artery (dpDMCA) flap of the hand: a novel DMCA-derived flap

**DOI:** 10.1007/s00238-017-1357-0

**Published:** 2017-09-04

**Authors:** Till Wagner, Oliver Kloeters, Dietmar Ulrich

**Affiliations:** Department of Hand, Plastic and Reconstructive Surgery, Radboud University Hospital, P.O. 9101, 6500 HB Nijmegen, The Netherlands

**Keywords:** Double pedicle, Dorsal-metacarpal-artery (DMCA) flap, Soft tissue defect

## Abstract

The dorsal-metacarpal-artery (DMCA) flap in its standard or extended version is considered as the working horse to cover dorsal soft tissue finger defects with exposed extensor tendon or bone. We hereby present a clinical case of an 80-year-old male patient who is right-handed and sustained a soft tissue defect of the proximal dorsal aspect of his left 5th finger and the postoperative outcome employing a modified transposition flap. The double-pedicled DMCA flap (dpDMCA flap) of the hand poses in adequate clinical scenarios a comparably fast and safe solution to cover dorsal finger defects extending just distal to the PIP joint. To the best of our knowledge, this is the first report of a DMCA-based flap with a double pedicle to cover soft tissue defects at the dorsum of the hand.

Level of Evidence: Level V, therapeutic study.

## Introduction

The dorsal-metacarpal-artery (DMCA) flap in its standard or extended version is considered as the working horse to cover dorsal soft tissue finger defects with exposed extensor tendon or bone. The vascular supply of the DMCA flap is since its first description [1, 2] and its modifications [3] described as deriving from one single perforating branch from the palmar aspect of the hand. Thus, with an absent or otherwise compromised perforator, the versatility of the basic DMCA flap rapidly becomes limited. We here describe a novel design of a DMCA-derived flap using two pedicles which in our view will broaden the reconstructive spectrum of dorsal finger defects.

## Case report

**Fig. 1:**
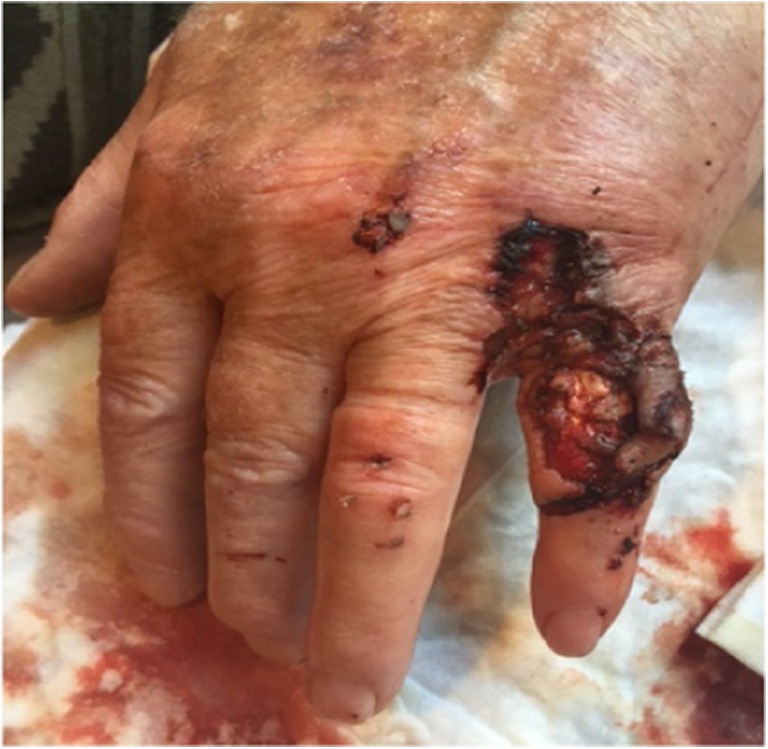
First appearance on clinical consultation at the emergency department

**Fig. 2:**
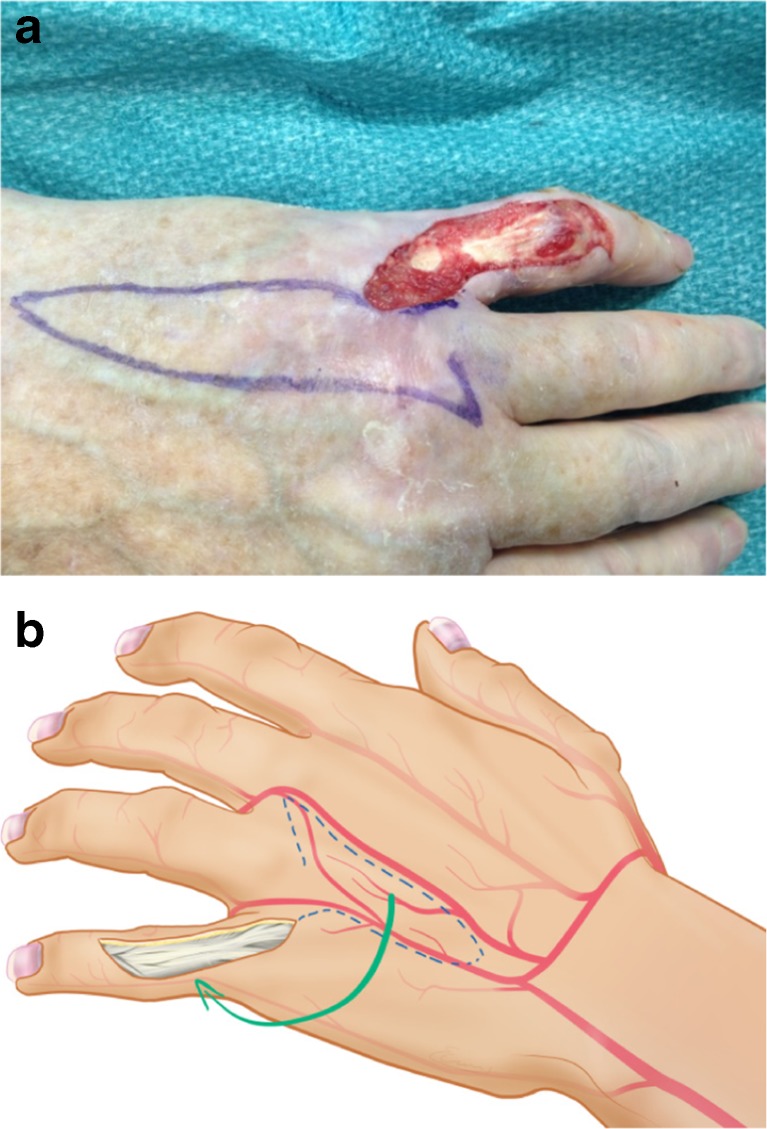
**a** Intraoperative view after thorough surgical debridement with demarcation of the dpDMCA flap. **b** Schematic representation of the proposed dpDMCA flap

**Fig. 3:**
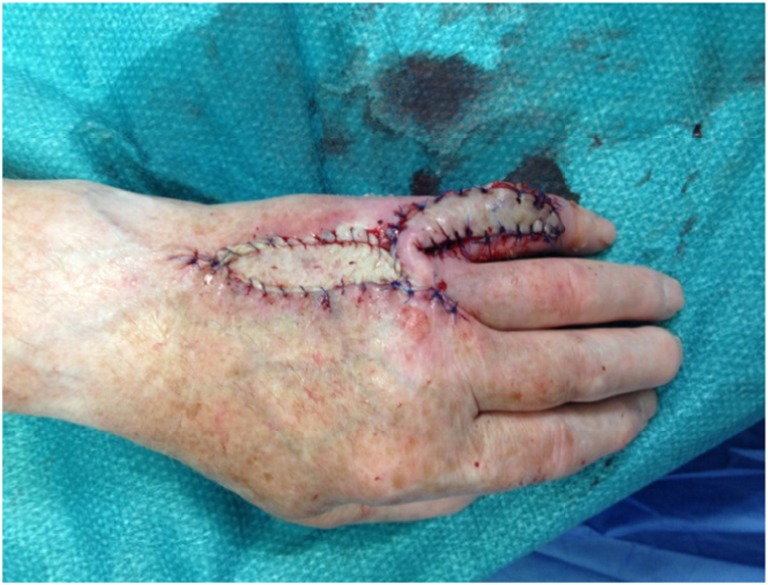
Immediate postoperative view showing the donor site closure by a full thickness skin graft

**Fig. 4:**
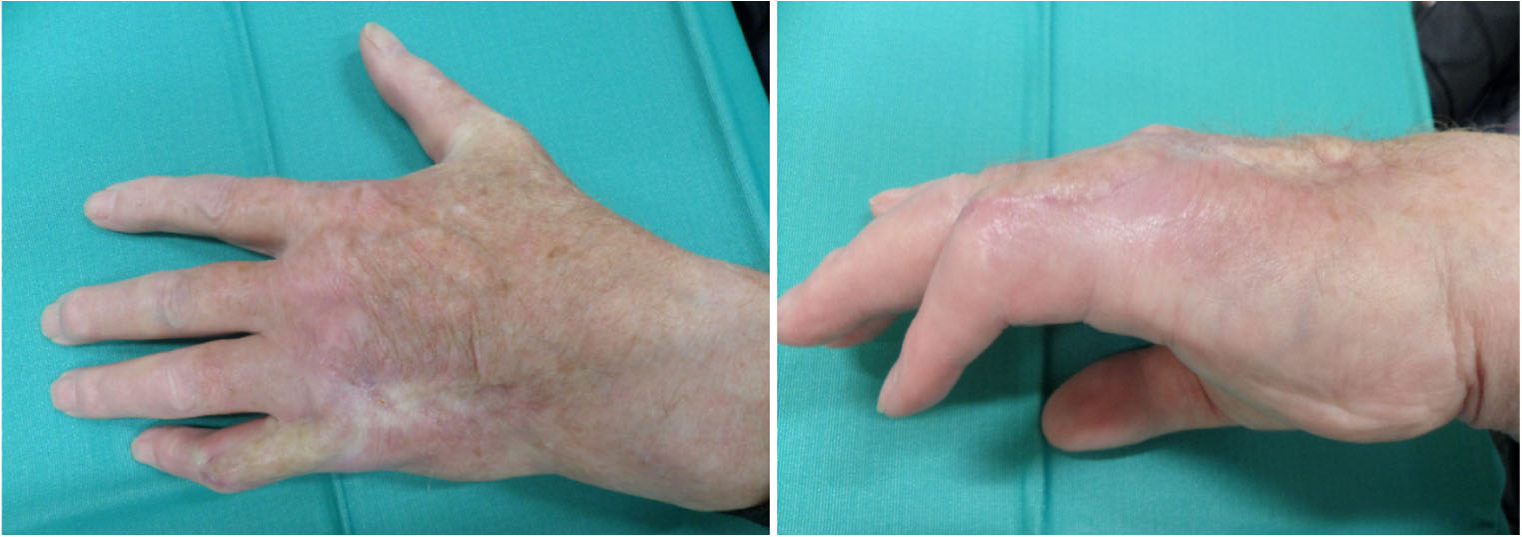
Postoperative view at 3 months

We present a case of an 80-year-old male patient with a traumatic laceration of his left pinky with intact extensor apparatus (Fig. 1), which was first treated by the ER department. On the 5th posttraumatic day, the patient presented to our department with partial necrosis and exposed extensor tendons. With an intact 4th web space perforator, the patient was scheduled for operation with a distally based DMCA-4 flap. After thorough debridement, only a weak signal at the level of the 4th web space perforator remained, and therefore we included the 3rd web space perforator into our transposition flap with an additional soft tissue island extension (Fig. 2a, b). Elevation of this flap started from proximal to distal in a standard fashion. When reaching the most distal perforator of the 3rd web space, we prepared this perforator with keeping the deep transverse ligament intact. This provided enough pivotal range to mobilize and rotate the flap into the defect. The donor site defect was closed by full thickness skin coverage (Fig. 3), and the wound was treated with cast immobilization for 3 weeks. The patient was satisfied with the result albeit a remaining flexion contracture of about 50 degrees (Fig. 4) resistant to further occupational therapy. After 6 months, range of motion was limited to PIP 0/50/90 and of his DIP joint 0/0/35.

## Discussion

Since the first description of the DMCA flap in 1990 [1, 2] and its later modification, the flap became a useful adjunct of the armamentarium for coverage of soft tissue finger defects [3]. In general, the donor site morbidity is low and the functional outcome is good to excellent [4]. Despite its ease of harvest and clinical application, the flap has its disadvantages since depending only on one single perforating palmar branch which can be involved in local crush injuries of the hand at times [5]. There still is ongoing discussion over safety and reliability of the 3rd and 4th web space perforators which serve as a vascular base of DMCA flaps. In early publications, the 3rd and the 4th intermetacarpal space has been seldom used as a donor site because of the previously reported variability in perforator presence [6, 7]. Recent publications tend to incline a notion advocating use of the DMCA flap all across the dorsum of the hand irrespectively of webspace location [8–10] along with the advice to allow for reperfusion prior to inset of the flap [11].

The variety of other options to reconstruct defects with DMCA-derived flaps are vast and were often tailored to the patients’ need. Reversed DMCA flaps can be harvested as fasciocutanous flaps, adipofascial flaps, digitometacarpal with an extended retrogade pedicle [12], or as composite flaps (combination of skin, adipofascia, tendon, and bone) [11, 13, 14], which are more technically demanding. Even free vascularized flaps are described [15] as well as delayed DMCA flaps [16] in smokers. Each of these flaps has advantages and disadvantages. The most relevant advantage of all is the possibility of a single stage procedure, usually low donor site morbidity and possibility for primary closure as well as tailored flap designs.

In this selected patient, we could demonstrate that our modified DMCA-derived transposition flap can serve as a useful adjunct to the aforementioned range of DMCA-modifications. The advantage of this procedure is a comparably short operating time (overall operation time 1 h 36 min) and a common recovery time for standard DMCA flaps (approx. 2 months). The patient was satisfied with the cosmetic outcome and functional restoration and no further treatment was desired or mandatory. Considering the flexion contracture, the patients’ first priority was to use his bicycle again and therefore denied any surgical suggestions such as teno-artholysis.

This double-pedicled flap may further be an option to develop other locoregional flaps to reconstruct more extensive soft tissue defects of the hand. We consider this combined flap of the dorsal aspect of the hand as a useful addition to the armamentarium of reconstructive options in selected cases but with presenting only one clinical case more patients with longer follow-up are required to confirm our findings.
